# Double Dose Reduction in the Equilibrium Phase of Chest-Pelvic CT With Low Tube Voltage and Forward-Projected Model-Based Iterative Reconstruction Solution

**DOI:** 10.7759/cureus.10545

**Published:** 2020-09-19

**Authors:** Shota Hosogoshi, Keiji Tada, Jun Iijima, Takafumi Kajitani, Rika Yoshida, Hajime Kitagaki

**Affiliations:** 1 Radiology, Shimane University Hospital, Izumo, JPN; 2 Radiology, Matsue City Hospital, Matsue, JPN; 3 Radiology, Faculty of Medicine, Shimane University, Izumo, JPN

**Keywords:** computed tomography, low-tube-voltage scanning, contrast medium reduction, radiation dose reduction, forward-projected model-based iterative reconstruction solution, low tube voltage

## Abstract

Objectives

This study aimed to examine whether a new imaging method (80-kV forward-projected model-based iterative reconstruction solution [FIRST] protocol) that uses a combination of low tube voltage and FIRST can reduce radiation dose and contrast medium volume by comparing the quality of the resulting image with that of the image obtained by 120-kV adaptive iterative dose reduction 3D protocol in the equilibrium phase of chest-pelvic computed tomography (CT).

Subjects and methods

Twenty-seven patients underwent CT by both protocols on different days. Two radiologists subjectively assessed image quality by scoring axial images for sharpness, contrast enhancement, noise, artifacts, and overall quality. The mean CT values, standard deviations, contrast-to-noise ratios, and signal-to-noise ratios in the liver, aorta, and erector spinae muscles were used for objective assessment. Radiation dose parameters included the CT dose index volume, dose-length product, effective dose, and size-specific dose estimate. Results were compared for different body mass index categories.

Results

The 80-kV FIRST protocol helped achieve mean reductions of 36.3%, 35.7%, and 36.6% in CT dose index volume, effective dose, and size-specific dose estimate, respectively (p < 0.01). Therefore, this protocol was regarded as comparable to the conventional protocol in image quality, except for visual sharpness.

Conclusions

The 80-kV FIRST protocol is capable of reducing radiation dose and contrast medium volume compared to the adaptive iterative dose reduction 3D protocol in the equilibrium phase of chest-pelvic CT.

## Introduction

To evaluate cancer recurrence, metastasis, and therapeutic effect, the equilibrium phase of chest-pelvic computed tomography (CT) is often used for cancer patients. Therefore, cancer patients are repeatedly exposed to radiation, and the use of contrast medium may increase their risk of developing contrast-induced nephropathy (CIN) [1-3]. Contrast-enhanced CT examination is considered to be a risk factor for CIN in patients with an estimated glomerular filtration rate (eGFR) of <30 mL/min/1.73 m^2^. Although it is reported that the risk for CIN is low in patients with an eGFR of ≥ 30 mL/min/1.73 m^2^, it is important to fully evaluate risk factors other than eGFR and to take preventive measures against CIN as necessary [4]. In particular, when reducing contrast medium as one of the preventive measures, a low tube voltage scanning is often executed to maintain the diagnostic capability [5].

Generally, it is said that a CT value of at least 50 Hounsfield units (HU) in liver parenchyma is required for the detection of lesions of metastatic liver tumors. In order to achieve this, it is necessary to administer an iodine amount of 520 mg/kg or more [6]. Yamashita et al. reported that it is necessary to administer 2.0-2.5 mL/kg of the 300 mg iodine/kg preparation to deliver the amount of contrast medium necessary for sufficiently dyeing the liver and portal vein [7].

Therefore, simply decreasing the contrast medium may not sufficiently secure the CT value necessary for diagnosis. On the other hand, since the CT value of iodine is higher in low tube voltage imaging, it is reported that even if the contrast medium is reduced to 60%, it has the same contrast effect as conventional imaging [8,9]. However, as the tube voltage decreases, the number of X-ray photons decreases in proportion to the square of tube voltage; therefore, image noise increases in low tube voltage scanning. Optimization of the standard deviation (SD) setting is important when using automatic exposure control (AEC) in low tube voltage scanning. This is because, in abdominal CT using AEC, under low scanning conditions such that an equivalent abdominal absorption dose is obtained, the noise level becomes higher as a result of low tube voltage scanning [10].

The clinical utility of the noise reduction effect in adaptive iterative dose reduction 3D (AIDR 3D) protocol due to the hybrid iterative reconstruction (IR) method is widely known. It was reported that using low tube voltage scanning and AIDR 3D protocol together would enable the reduction of exposure dose and contrast medium volume without compromising image quality when compared with conventional tube voltage scanning [11,12]. However, it is suggested that AIDR 3D images may change in sharpness depending on the subject’s physique, and image quality using the IR method requires verification regarding the physique of the patient [13].

Recently, forward-projected model-based IR solution (FIRST), a type of full IR method, has been developed to further improve image quality in CT. FIRST is an algorithm that can obtain optimum image quality with little image noise and few artifacts even with low-dose projection data by using various models in the process of repeating back projection and forward projection. As a result, FIRST can be expected to reduce the needed radiation dose without compromising image quality when compared with AIDR 3D protocol. However, currently, clinical investigation using low tube voltage scanning and the full IR method in combination is not common.

Therefore, in this study, we used two protocols: (1) 120-kV AIDR 3D protocol, in which a normal dose of a contrast medium was administered, the subject was scanned at a conventional tube voltage, and image reconstruction was performed with AIDR 3D protocol, and (2) 80-kV FIRST protocol, in which contrast medium was reduced to 60%, the subject was scanned at low tube voltage, and image reconstruction was performed with FIRST. The subjects were patients with reduced renal function, and these patients were categorized by body mass index (BMI) and compared. We examined whether the 80-kV FIRST protocol is capable of reducing radiation dose and the amount of contrast medium without compromising image quality compared with the 120-kV AIDR 3D protocol.

## Materials and methods

Institutional Review Board approval was obtained for this study. Furthermore, we obtained prior written informed consent from all patients participating in this study.

Study population

This study included 27 patients (19 men and 8 women) who underwent contrast-enhanced CT examination at our hospital from December 2016 to August 2018. These patients had an eGFR of <60 mL/min/1.73 m^2^ within three months of the contrast-enhanced CT examination date, and when the radiologist judged that contrast medium reduction was necessary, their contrast-enhanced CT examination was performed with the 80-kV FIRST protocol. Furthermore, these patients underwent a contrast-enhanced CT examination with the 120-kV AIDR 3D protocol if they had an eGFR of ≥ 60 mL/min/1.73 m^2^ within three months of the contrast-enhanced CT examination date. The median interval between the two scans was six months (range: 0-17 months). The mean age of the patients was 74.1 ± 10.5 years (range: 52-92 years) at the time of the 120-kV AIDR 3D protocol and 74.5 ± 10.5 years (range: 52-93 years) at the time of the 80-kV FIRST protocol. Patients were divided into three BMI categories: <20 (n = 7), 20-24.9 (n = 15), and ≥25 (n = 5). There was no significant difference in scan acquisition length between the 120-kV AIDR 3D protocol and the 80-kV FIRST protocol (724.4 ± 52.1 mm and 728.5 ± 47.2 mm, respectively; p = 0.17).

Scan protocol and image reconstruction

All patients were examined on a 320-row CT scanner (Aquilion ONE ViSION Edition, Canon Medical Systems, Otawara, Japan). The scout view was scanned only in the anteroposterior direction. AEC (SURE Exposure 3D, Canon Medical Systems) was used to determine tube current. The noise index setting was based on the filtered back-projection (FBP) image with a slice thickness of 5 mm, with SD set to 13 HU for the 120-kV AIDR 3D protocol and to 19 HU for the 80-kV FIRST protocol. The remaining scan parameters for both these protocols were as follows: detector configuration of 80 × 0.5 mm (detector collimation), gantry rotation time of 0.5 seconds, and helical pitch (beam pitch) of 0.813.

Contrast injection was delivered over the course of 50 seconds with the 120-kV AIDR 3D protocol (550 mg iodine/kg) and 80-kV FIRST protocol (330 mg iodine/kg) by power injector (Dual Shot GX 7, Nemoto Kyorindo, Tokyo, Japan). In both protocols, we scanned from the chest to the pelvis 50 seconds after completing the injection of the contrast medium.

In all examinations, ioversol (240 mg iodine/mL in a 100-mL syringe [Optiray 240, Fuji-Pharma, Tokyo, Japan] and 350 mg iodine/mL in a 135-mL syringe [Optiray 350, Fuji-Pharma]), iopromide (300 mg iodine/mL in a 100-mL syringe [Iopromide 300, FUJIFILM Toyama Chemical, Tokyo, Japan] and 370 mg iodine/mL in a 100-mL syringe [Iopromide 370, FUJIFILM Toyama Chemical), iohexol (300 mg iodine/mL in 100-mL syringe [Omnipaque 300, Daiichi-Sankyo, Tokyo, Japan] and 350 mg iodine/mL in 100-mL syringe [Omnipaque 350, Daiichi-Sankyo]), or iomeprol (350 mg iodine/mL in 100- and 150-mL syringes [Iomeron 350, Eisai, Tokyo, Japan]) were used as contrast medium. The contrast medium administered to the patient was determined from among these by the amount of iodine specified in the protocol and patient's weight.

Display FOV (field of view) was set according to the physique of the patient. The image slice thickness and slice interval were reconstructed to 5 mm. In the 120-kV AIDR 3D protocol, the FC04 reconstruction kernel and AIDR 3D mild algorithm, which is Hybrid IR, were used for image reconstruction. In the 80-kV FIRST protocol, the FIRST Body sharp standard, Full IR algorithm was used for image reconstruction. Table 1 shows the scan and contrast medium parameters of the 120-kV AIDR 3D and 80-kV FIRST protocols.

**Table 1:** Scan and contrast medium parameters of 120-kV AIDR 3D and 80-kV FIRST protocols ^1^The tube currents were determined by automatic exposure control. ^2^The noise index was set at a slice thickness of 5 mm. AIDR 3D, Adaptive Iterative Dose Reduction 3D (Canon Medical Systems); FIRST, Forward-projected model-based Iterative Reconstruction SoluTion (Canon Medical Systems)

	120-kV AIDR 3D protocol	80-kV FIRST protocol
Detector collimation (mm)	0.5 × 80	0.5 × 80
Slice/interval thickness (mm)	5.0/5.0	5.0/5.0
Gantry rotation time (ms)	500	500
Tube voltage (kV)	120	80
Tube current (mA)^1^	50-580	50-680
Noise index (HU)^2^	13	19
Beam pitch	0.813	0.813
Reconstruction algorithm	AIDR 3D mild	FIRST Body sharp standard
Reconstruction kernel	FC04	-
Contrast media dose (mg iodine/kg)	550	330
Injection duration (sec)	50	50
Scan delay (sec)	50	50

Estimation of the radiation dose

The volume CT dose index (CTDIvol) and dose-length product (DLP) generated on dose reports by the scanner were recorded for each examination.

Effective dose (ED) was calculated according to the following calculation formula published in Publication 102 of the International Commission on Radiological Protection (ICRP) [14]:

ED [mSv] = DLP [mGy･cm] × k [mSv･mGy-1･cm-1] (1)

The coefficient k is shown for each age group and scanned body part in the ICRP Publication 102. In this study, the coefficient k was set to 0.015 in all cases.

Size-specific dose estimates (SSDEs) were calculated by multiplying CTDIvol by a conversion factor. The conversion factor was determined from the effective diameter calculated from the anteroposterior and lateral lengths measured from the axial image. Furthermore, the conversion factor was based on the TG 204 report of the American Association of Physicists in Medicine (AAPM), and its value in the 32 cm phantom was used [15]. Measurements of anteroposterior and lateral lengths were performed by the same radiological technologist using slices at the origin of the celiac artery.

Subjective assessment of image quality

Two board-certified radiologists independently reviewed all axial slices of the 5-mm reconstructed equilibrium phase images, randomly and blinded to both protocols.

They used a four-point subjective scale to grade sharpness (1, unacceptable; 2, acceptable; 3, good; 4, excellent), contrast enhancement (1, unacceptable; 2, acceptable; 3, good; 4, excellent), image noise (1, present and unacceptable; 2, present and interfering with adjacent structures; 3, present but not interfering with adjacent structures; 4, minimal), artifacts (1, present and unacceptable; 2, present and interfering with adjacent structures; 3, present but not interfering with adjacent structures; 4, minimal), and overall image quality (1, unacceptable; 2, acceptable; 3, good; 4, excellent).

The 3D image analysis system "SYNAPSE VINCENT" (FUJIFILM Corporation, Tokyo, Japan) was used to view all scans. Patient information and scan parameters were anonymized. The observation time was unlimited. They were asked to observe several examples of the 120-kV AIDR 3D protocol and the 80-kV FIRST protocol beforehand as training on the scoring system and they began subjective assessment after the training.

Objective assessment of image quality

**Figure 1:**
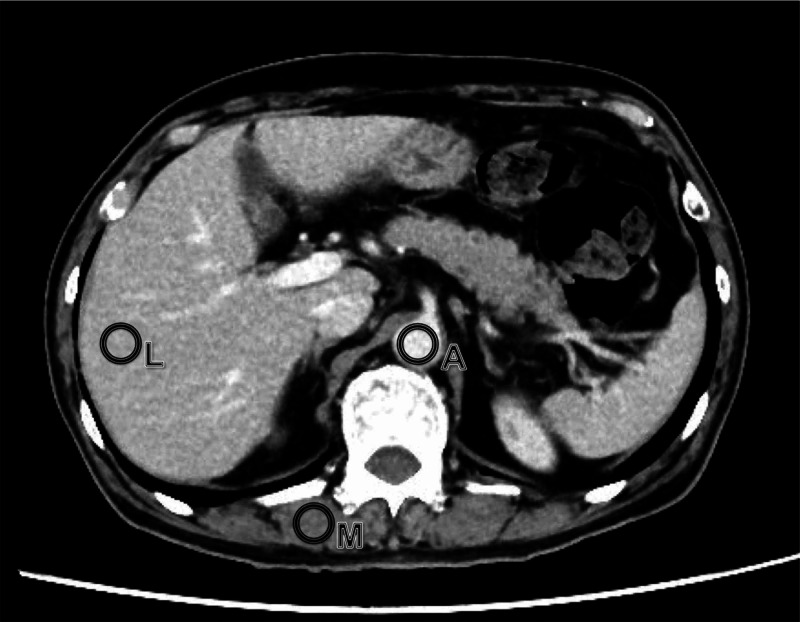
CT scan of a 69-year-old man with bladder cancer This figure is an axial image of the beginning of the celiac artery in the 80-kV FIRST protocol. The ROIs were placed in aorta (A), liver (L), and the erector spinae muscle (M). CT, computed tomography; FIRST, forward-projected model-based iterative reconstruction solution; ROI, region of interest

Objective assessment of image quality was performed by one radiological technologist. The mean CT values (HU) and SDs of the liver, aorta, and erector spinae muscles were measured using slices at the origin of the celiac artery for all examinations. Image J (version 1.48u, NIH) was used for measurement, and a circular region of interest (ROI) was manually placed as shown in Figure 1 [16,17]. The ROI placed in the liver avoided blood vessels and lesions, and the ROI placed in the aorta avoided the aortic wall. The signal-to-noise ratio (SNR) and contrast-to-noise ratio (CNR) of the liver and aorta were calculated from the mean CT value and SD with the following formula:

SNR = ROI / SD (2)

CNR = (ROI - ROIm) / SDm (3)

In the preceding equation, ROI is the mean CT value of the organ of interest, SD is the SD of the CT value in the organ of interest, ROIm is the mean CT value of the erector spinae muscles, and SDm is the SD of the CT value of the erector spinae muscles. Furthermore, a figure of merit (FOM) was calculated as the ratio of the CNR2 and the CTDIvol. This was used because it can associate image quality with radiation dose [18].

Statistical analysis

Numerical data were expressed as the mean ± SD. All subjective and objective assessments of image quality obtained under the 120-kV AIDR 3D protocol and the 80-kV FIRST protocol were compared. Patients were divided into three BMI categories to assess radiation exposure and image quality for each physique: <20 (n = 7), 20-24.9 (n = 15), and ≥25 (n = 5). Differences in the mean values between the two protocols’ data in the objective assessment of image quality were determined using the paired t-test. Differences in the median values between the two protocols’ data in the subjective assessment of image quality were determined using the Wilcoxon signed-rank test. A p-value of <0.05 was considered to indicate a statistically significant difference. Data were analyzed using R (version 3.0.2, R Foundation for Statistical Computing).

## Results

Radiation dose

Table 2 shows the CTDIvol, DLP, ED, and SSDE values for each protocol. All radiation dose parameters were significantly lower for the 80-kV FIRST protocol compared with those in the 120-kV AIDR 3D protocol for the entire study population and also when the patients were divided according to BMI category (p < 0.01). The mean reduction in CTDIvol was 41.2% for patients with a BMI of <20, 32.1% for patients with a BMI of 20-24.9, 43.2% for patients with a BMI of ≥25, and 36.3% for all patients. For DLP and ED, mean reductions were 40.5% for patients with a BMI of <20, 31.0% for patients with a BMI of 20-24.9, 42.5% for patients with a BMI of ≥25, and 35.7% for all patients. For SSDE, mean reductions were 40.7% for patients with a BMI of <20, 32.4% for patients with a BMI of 20-24.9, 43.1% for patients with a BMI of ≥25, and 36.6% for all patients.

**Table 2:** Radiation dose parameters of chest-pelvic CT examinations reconstructed with AIDR 3D and FIRST protocols Note: data are presented as mean ± standard deviation ^a^Paired t-test BMI, body mass index (weight in kilograms divided by the square of height in meters); AIDR 3D, Adaptive Iterative Dose Reduction 3D (Canon Medical Systems); FIRST, Forward-projected model-based Iterative Reconstruction SoluTion (Canon Medical Systems); CTDIvol, volume computed tomography dose index; DLP, dose-length product; ED, effective dose; SSDE, size-specific dose estimate

Radiation dose parameters	All patients (n = 27)			BMI < 20 (n = 7)			BMI 20-24.9 (n = 15)			BMI ≥ 25 (n = 5)			
	120-kV AIDR 3D	80-kV FIRST	p-Value^a^	120-kV AIDR 3D	80-kV FIRST	p-Value^a^	120-kV AIDR 3D	80-kV FIRST	p-Value^a^		120-kV AIDR 3D	80-kV FIRST	p-Value^a^
CTDI_vol _(mGy)	13.5 ± 3.2	8.6 ± 2.3	<0.01	9.9 ± 1.5	5.8 ± 1.4	<0.01	13.7 ± 2.3	9. 3 ± 1.3	<0.01		17.6 ± 1.3	10.0 ± 2.3	<0.01
DLP (mGy･cm)	1044.3 ± 288.0	671.3 ± 193.1	<0.01	766.0 ± 153.9	455.9 ± 121.4	<0.01	1050.6 ± 213.1	724.5 ± 128.0	<0.01		1415.1 ± 179.9	813.1 ± 194.7	<0.01
ED (mSv)	15.7 ± 4.3	10.1 ± 1.9	<0.01	11.5 ± 2.3	6.8 ± 1.8	<0.01	15.8 ± 3.2	10.9 ± 1.9	<0.01		21.2 ± 2.7	12.2 ± 2.9	<0.01
SSDE (mGy)	20.2 ± 3.3	12.8 ± 2.8	<0.01	16.2 ± 1,6	9.6 ± 1.6	<0.01	20.7 ± 2.6	14.0 ± 1.5	<0.01		23.9 ± 0.8	13.6 ± 3.5	<0.01

Subjective assessment of image quality

**Figure 2:**
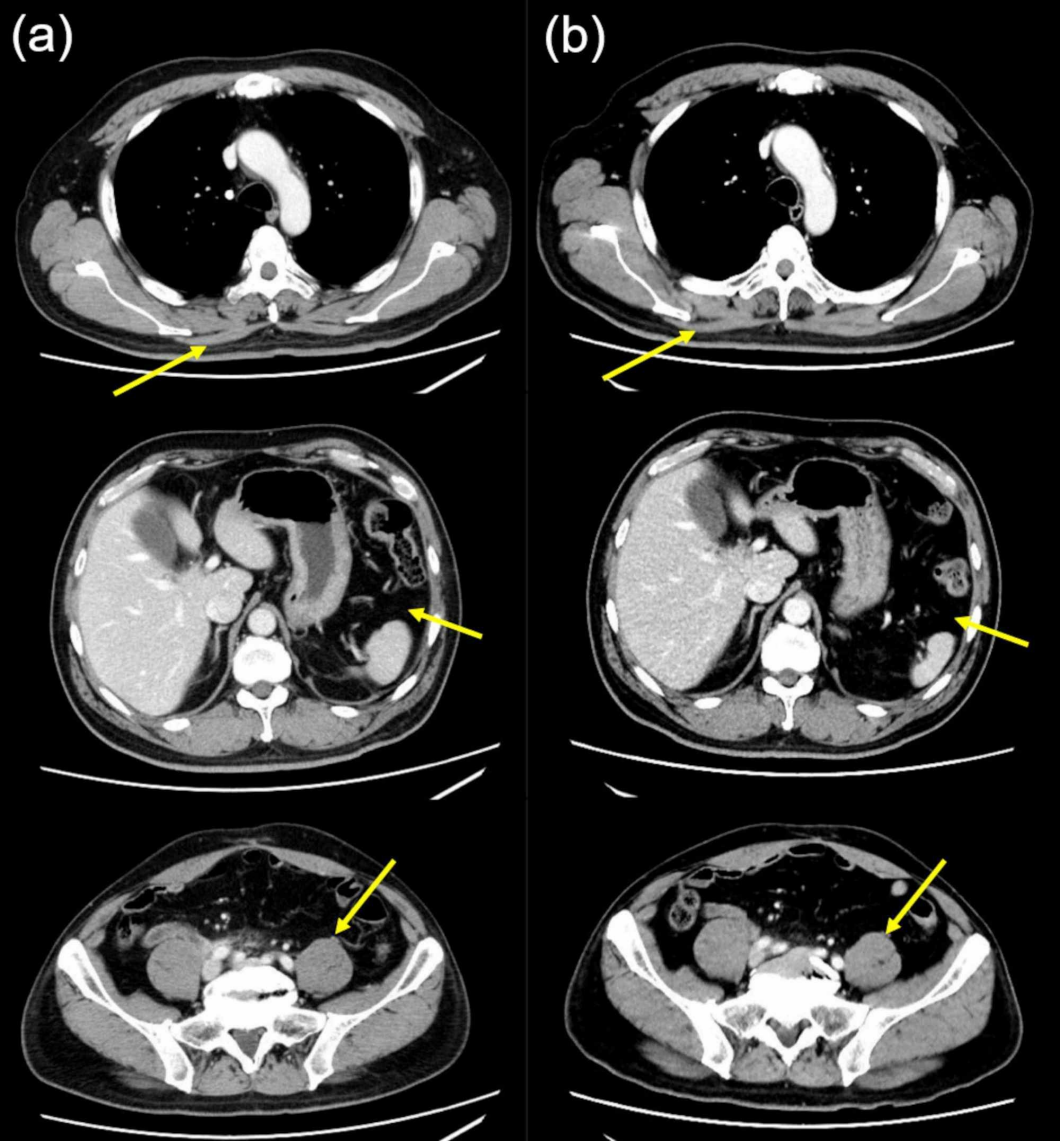
CT scan of a 55-year-old man (body weight, 80.0 kg; body mass index, 26.1) with postoperative bladder cancer (a) A series of axial images reconstructed by AIDR 3D in January 2017 after injecting a contrast medium (550 mg iodine/kg) and scanning the equilibrium phase of the chest-pelvic computed tomography (CT) at 120 kV. (b) A series of axial images reconstructed by FIRST in May 2017 after injecting a contrast medium (330 mg iodine/kg) and scanning the equilibrium phase of the chest-pelvic CT at 80 kV. (a and b) Top image shows the chest, middle image shows the superior abdomen, and bottom image shows the pelvis. The visual score was good for all (a) and (b) from all the observers in contrast enhancement, image noise, artifacts, and overall image quality. However, sharpness in (b) was inferior to all observers in comparison to (a). Areas with low contrast, such as the muscles and fats indicated by the arrows, were rated particularly poor sharpness. The effective dose was reduced by 34.9% (24.9 mSv to 16.2 mSv) by scanning with 80-kV FIRST as opposed to 120-kV AIDR 3D, and the size-specific dose estimate was reduced by 33.6% (25.0 mGy to 16.6 mGy). CT, computed tomography; AIDR 3D, adaptive iterative dose reduction 3D; FIRST, forward-projected model-based iterative reconstruction solution

Table 3 shows the subjective assessment of image quality for each protocol. The visual score in sharpness was significantly higher with the 120-kV AIDR 3D protocol in each BMI category (p < 0.01). The visual score in artifacts was significantly higher with the 120-kV AIDR 3D protocol in patients with a BMI of ≥25 (p = 0.03). There was no significant difference in the visual scores for contrast enhancement and overall image quality between the 120-kV AIDR 3D protocol and the 80-kV FIRST protocol. Representative cases are shown in Figure 2.

**Table 3:** Subjective assessment of image quality of chest-pelvic CT examinations reconstructed with AIDR 3D and FIRST protocols Note: data are presented as mean ± standard deviation ^a^Wilcoxon signed-rank test BMI, body mass index; AIDR 3D, Adaptive Iterative Dose Reduction 3D (Canon Medical Systems); FIRST = Forward-projected model-based Iterative Reconstruction SoluTion (Canon Medical Systems); CT, computed tomography.

Subjective parameters	All patients (n = 27)			BMI < 20 (n = 7)			BMI 20-24.9 (n = 15)			BMI ≥ 25 (n = 5)		
	120-kV AIDR 3D	80-kV FIRST	p-Value^a^	120-kV AIDR 3D	80-kV FIRST	p-Value^a^	120-kV AIDR 3D	80-kV FIRST	p-Value^a^	120-kV AIDR 3D	80-kV FIRST	p-Value^a^
Sharpness	3.1 ± 03	2.4 ± 0.5	<0.01	3.1 ± 0.3	2.3 ± 0.5	<0.01	3.2 ± 0.4	2.4 ± 0.5	<0.01	3.1 ± 0.3	2.4 ± 0.5	0.01
Contrast enhancement	2.8 ± 0.6	2.9 ± 0.6	0.23	2.9 ± 0.5	3.1 ± 0.6	0.41	2.8 ± 0.6	2.9 ± 0.6	0.40	2.6 ± 0.5	2.7 ± 0.6	0.82
Image noise	3.8 ± 0.4	3.5 ± 0.5	<0.01	3.8 ± 0.4	3.5 ± 0.5	0.07	3.7 ± 0.4	3.6 ± 0.5	0.07	3.7 ± 0.5	3.4 ± 0.7	0.15
Artifacts	3.5 ± 0.7	3.3 ± 0.7	0.10	3.5 ± 0.7	3.4 ± 0.7	0.79	3.5 ± 0.7	3.5 ± 0.6	0.85	3.7 ± 0.5	3.0 ± 0.6	0.03
Overall image quality	3.1 ± 0.5	3.0 ± 0.3	0.21	3.2 ± 0.4	3.0 ± 0.4	0.23	3.0 ± 0.5	3.1 ± 0.2	0.78	3.2 ± 0.4	2.8 ± 0.4	0.07

Objective assessment of image quality

Table 4 shows the objective assessment of image quality for each protocol. There were no significant differences in the mean CT values of the liver, aorta, and erector spinae muscle when compared between the 120-kV AIDR 3D protocol and the 80-kV FIRST protocol. Image noise in the aorta was significantly higher with the 80-kV FIRST protocol in patients with a BMI of ≥25 (p = 0.01). The liver SNR was significantly higher with the 120-kV AIDR 3D protocol for all patients (p = 0.03). There were no significant differences in the liver or aorta CNRs between the 120-kV AIDR 3D protocol and the 80-kV FIRST protocol. The FOM was significantly higher with 80-kV FIRST in all patients’ livers (p = 0.05) and aortae (p < 0.01), and in the aortae of patients with a BMI of 20-24.9 (p < 0.01).

**Table 4:** Objective assessment of image quality of chest-pelvic CT examinations reconstructed with AIDR 3D and FIRST protocols Note: data are presented as mean ± standard deviation ^a^Paired t-test AIDR 3D, Adaptive Iterative Dose Reduction 3D (Canon Medical Systems); FIRST, Forward-projected model-based Iterative Reconstruction SoluTion (Canon Medical Systems); CT, computed tomography

Objective parameters, organ	All patients (n = 27)			BMI < 20 (n = 7)			BMI 20-24.9 (n = 15)			BMI ≥ 25 (n = 5)		
	120-kV AIDR 3D	80-kV FIRST	p-Value^a^	120-kV AIDR 3D	80-kV FIRST	p-Value^a^	120-kV AIDR 3D	80-kV FIRST	p-Value^a^	120-kV AIDR 3D	80-kV FIRST	p-Value^a^
Mean CT value (HU)												
Liver	106.3 ± 15.5	105.5 ± 14.0	0.73	110.8 ± 9.3	109.4 ± 9.5	0.82	105.6 ± 17.9	105.9 ± 15.4	0.93	102.1 ± 13.0	98.7 ± 12.4	0.54
Aorta	146.7 ± 23.0	149.0 ± 18.8	0.47	149.6 ± 14.7	150.4 ± 15.5	0.93	147.6 ± 24.5	148.5 ± 17.9	0.81	146.1 ± 19.3	148.6 ± 24.7	0.47
Muscle	65.1 ± 6.1	65.4 ± 7.9	0.75	63.1 ± 6.1	61.1 ± 4.6	0.30	65.3 ± 6.6	66.3 ± 8.5	0.43	67.1 ± 3.0	68.6 ± 6.9	0.65
Image noise (HU)												
Liver	9.5 ± 1.1	10.0 ± 1.2	0.07	8.9 ± 0.6	9.4 ± 0.8	0.36	9.3 ± 1.0	9.6 ± 0.8	0.50	10.7 ± 1.2	11.9 ± 0.3	0.09
Aorta	10.7 ± 1.1	11.2 ± 1.7	0.12	10.4 ± 0.8	11.1 ± 1.4	0.25	10,6 ± 1.0	10.8 ± 1.8	0.74	11.6 ± 1.3	12.7 ± 1.2	0.01
Muscle	11.2 ± 1.7	11.5 ± 1.5	0.54	11.3 ± 1.3	11.9 ± 1.8	0.46	11.3 ± 2.0	11.0 ± 1.3	0.43	10.8 ± 1.1	12.4 ± 0.9	0.15
Signal-to-noise ratio												
Liver	11.4 ± 2.2	10.8 ± 2.0	0.03	12.5 ± 0.8	11.8 ± 1.5	0.28	11.5 ± 2.4	11.1 ± 1.7	0.32	9.7 ± 1.6	8.3 ± 1.0	0.16
Aorta	13.8 ± 2.7	13.6 ± 2.8	0.60	14.6 ± 2.0	13.7 ± 2.1	0.46	14.0 ± 2.6	14.2 ± 2.9	0.68	12.4 ± 3.3	11.9 ± 2.5	0.32
Contrast-to-noise ratio												
Liver	3.7 ± 1.4	3.5 ± 1.5	0.55	4.3 ± 0.9	4.2 ± 1.4	0.93	3.6 ± 1.6	3.6 ± 1.5	0.99	3.3 ± 1.2	2.4 ± 1.1	0.11
Aorta	7.3 ± 1.8	7.4 ± 2.0	0.76	7.8 ± 1.6	7.7 ± 2.0	0.94	7. 2 ± 1.8	7.5 ± 1.8	0.50	6.7 ± 2.2	6.6 ± 2.5	0.84
Figure of merit												
Liver	1.2 ± 1.0	1.7 ± 1.6	0.05	1.8 ± 1.1	2.7 ± 1.1	0.25	1.1 ± 1.0	1.6 ± 1.7	0.13	0.5 ± 0.3	0.6 ± 0.4	0.73
Aorta	4.1 ± 2.8	6.4 ± 3.7	<0.01	6.0 ± 3.9	9.1 ± 3.3	0.14	3.7 ± 1.9	5.7 ± 3.1	<0.01	2.4 ± 1.3	4.7 ± 3.9	0.23

## Discussion

To our knowledge, there is no report comparing full IR in low tube voltage scanning and hybrid IR in normal tube voltage scanning for the purpose of reducing contrast medium. Comparison of FBP and hybrid IR accounts for the majority of past studies in the reduction of contrast medium by low tube voltage scanning. However, at present, hybrid IR is the mainstream image reconstruction of the trunk region, and the comparison between FBP and FIRST is different from the current clinical practice; therefore, we compared AIDR 3D and FIRST in this study. The 80-kV FIRST protocol in all BMI categories maintained an image quality roughly equivalent to that of the 120-kV AIDR 3D protocol, and the radiation dose was significantly reduced. Furthermore, our findings validated previous reports that an equivalent contrast effect was obtained even if the contrast medium was reduced to 60% by reducing the tube voltage from 120 kV to 80 kV [8,9].

We conducted preliminary phantom experiments and adjusted the AEC so that the image resulting from the 120-kV AIDR 3D protocol and the image from the 80-kV FIRST protocol have equivalent SDs. As a result, the 80-kV FIRST protocol allowed the AEC settings to be set lower than those for the 120-kV AIDR 3D protocol. This resulted in a radiation dose reduction with the 80-kV FIRST protocol.

Meanwhile, there was a difference of about 10% in the radiation dose reduction rate between the category of BMI of 20-24.9 and the other BMI categories. This factor seems to be due to the radiation dose shortage in patients with BMI ≥ 25 in the 80-kV FIRST protocol, which caused the radiation dose reduction rate to increase compared to patients with a BMI of 20-24.9.

Therefore, it is difficult to determine a dose to satisfy the AEC setting in patients with BMI ≥ 25 in the 80-kV FIRST protocol. However, in this study, the 80-kV FIRST protocol did not cause any degradation in objective image quality in any of the BMI categories.

In contrast, the 80-kV FIRST protocol in all BMI categories significantly decreased subjective image sharpness compared to the 120-kV AIDR 3D protocol. Comparing the image quality of FIRST and AIDR 3D, Maeda et al. reported that FIRST was superior for image noise and sharpness in coronary CT angiography [19]. Wu et al. also reported that in abdominal CT angiography, the FIRST algorithm improved the visualization of small blood vessels such as the hepatic artery [20]. Therefore, the FIRST algorithm can obtain higher spatial resolution than the AIDR 3D algorithm when only blood vessels in the arterial phase are targeted.

However, this study is directed to the equilibrium phase, and the contrast is different between the arterial phase and the equilibrium phase; therefore, the results on sharpness were considered different. In other words, since structures with various contrasts exist in the equilibrium phase image, it is possible that an observer could detect a change in sharpness due to a difference in contrast that occurs in the FIRST algorithm. For example, the edge of a low contrast area such as fat or muscle gives an impression that sharpness is lower than that in a high contrast area such as a bone or a blood vessel. In addition, the IR algorithm changes the noise reduction effect according to the excess or deficiency of the radiation dose at the time of acquiring the projection data, and the sharpness decreases as the noise reduction acts strongly [21].

Therefore, dose and contrast dependency in the FIRST algorithm are factors that gave our observer the impression of impaired sharpness. Nevertheless, the contrast enhancement and overall image quality are equivalent between the two protocols, and we believe that the sharpness reduction to the observer is not a big problem.

As a limitation of this study, there are few subjects in the category of BMI ≥ 25. Furthermore, we did not evaluate the diagnostic accuracy of the CT examinations, which would be a more appropriate approach in specific clinical contexts (e.g., detection of metastasis in patients with cancer).

## Conclusions

The 80-kV FIRST protocol was capable of reducing radiation dose and amount of contrast medium when compared to the 120-kV AIDR 3D protocol in the equilibrium phase of chest-pelvic CT.
